# Ending preventable stillbirths and improving bereavement care: a scorecard for high- and upper-middle income countries

**DOI:** 10.1186/s12884-023-05765-5

**Published:** 2023-06-30

**Authors:** Esti Charlotte de Graaff, Susannah Hopkins Leisher, Hannah Blencowe, Harriet Lawford, Jillian Cassidy, Paul Richard Cassidy, Elizabeth S. Draper, Alexander E. P. Heazell, Mary Kinney, Paula Quigley, Claudia Ravaldi, Claire Storey, Alfredo Vannacci, the E. P. S. in High-Resource Countries Scorecard Collaboration Group, Paul Corcoran, Robin Cronin, Jan Jaap Erwich, Mika Gissler, Sanne Gordijn, Guilherme Ramires de Jesús, Jeannette Klimont, Aline Lecomte, Marzia Loghi, Margaret Murphy, Urelija Rodin, Guy Weber, Lindsey Wimmer, Vicki Flenady

**Affiliations:** 1 Mater Research Institute, NHMRC Centre of Research Excellence in Stillbirth, University of Queensland, Brisbane, Australia; 2 International Stillbirth Alliance, Millburn, USA; 3 University of Utah School of Medicine, Salt Lake City, USA; 4 Centre for Maternal, Adolescent, Reproductive and Child Health, London School of Hygiene & Tropical Medicine, London, UK; 5 Asociación Umamanita, Girona, Spain; 6 MBRRACE-UK, Department of Population Health Sciences, University of Leicester, Leicester, UK; 7 Maternal and Fetal Health Research Centre, Division of Developmental Biology and Medicine, School of Medical Sciences, Faculty of Biology, Medicine and Health, University of Manchester, Manchester, UK; 8 School of Public Health, University of the Western Cape, Belville, South Africa; 9 PeaRL Perinatal Research Laboratory, CiaoLapo Foundation for Perinatal Health, Department of Neurosciences, Psychology, Drug Research and Child Health, University of Florence, Florence, Italy

**Keywords:** Stillbirth, High-income countries, High-resource setting, Scorecard, Equity, Stigma, Data, Performance indicators, Accountability, Bereavement

## Abstract

**Background:**

Despite progress, stillbirth rates in many high- and upper-middle income countries remain high, and the majority of these deaths are preventable. We introduce the Ending Preventable Stillbirths (EPS) Scorecard for High- and Upper Middle-Income Countries, a tool to track progress against the Lancet’s 2016 EPS Series Call to Action, fostering transparency, consistency and accountability.

**Methods:**

The Scorecard for EPS in High- and Upper-Middle Income Countries was adapted from the Scorecard for EPS in Low-Income Countries, which includes 20 indicators to track progress against the eight Call to Action targets. The Scorecard for High- and Upper-Middle Income Countries includes 23 indicators tracking progress against these same Call to Action targets. For this inaugural version of the Scorecard, 13 high- and upper-middle income countries supplied data. Data were collated and compared between and within countries.

**Results:**

Data were complete for 15 of 23 indicators (65%). Five key issues were identified: (1) there is wide variation in stillbirth rates and related perinatal outcomes, (2) definitions of stillbirth and related perinatal outcomes vary widely across countries, (3) data on key risk factors for stillbirth are often missing and equity is not consistently tracked, (4) most countries lack guidelines and targets for critical areas for stillbirth prevention and care after stillbirth and have not set a national stillbirth rate target, and (5) most countries do not have mechanisms in place for reduction of stigma or guidelines around bereavement care.

**Conclusions:**

This inaugural version of the Scorecard for High- and Upper-Middle Income Countries highlights important gaps in performance indicators for stillbirth both between and within countries. The Scorecard provides a basis for future assessment of progress and can be used to help hold individual countries accountable, especially for reducing stillbirth inequities in disadvantaged groups.

**Supplementary Information:**

The online version contains supplementary material available at 10.1186/s12884-023-05765-5.

## Key messages

1. Wide disparities in stillbirth rates exist between and within high- and upper-middle income countries, indicating that further reduction in stillbirth rates is possible.

The existence of disparities in stillbirth rates across high- and upper-middle income countries shows that further improvements in stillbirth prevention are possible and makes the continued reduction of stillbirth rates a public health imperative. This includes balanced measures to ensure that stillbirth prevention strategies do not result in an increase in related perinatal outcomes, such as preterm birth and neonatal death.

2. Numerous disparate national stillbirth definitions are used in high- and upper-middle income countries, limiting comparisons necessary to drive change.

The use of multiple definitions for stillbirth and related perinatal outcomes makes it difficult to assess and compare individual countries’ performance, and to use data for advocacy and accountability for stillbirth prevention and support. Universal definitions specific to high- and upper-middle income countries should be developed and used.

3. Data on key risk factors and equity in stillbirth rates are limited, however, underline the need for increased focus on the most affected communities.

Tracking stillbirth rates associated with risk factors such as socio-economic deprivation and adolescent, or unplanned pregnancies can help in the development, adaptation and implementation of stillbirth prevention strategies. Yet these data are often lacking. Similarly, data on equity in stillbirth and related birth outcomes can help to inform stillbirth prevention strategies; however, such data are frequently unavailable, contributing to insufficient targeting of at-risk populations. National equity targets for birth outcome are required.

4. Most high- and upper-middle income countries lack guidelines and targets on key areas critical for stillbirth prevention and care after stillbirth, including national stillbirth rate targets.

The absence of guidelines and targets on key areas critical for stillbirth prevention and care after stillbirth means healthcare providers, civil society and other stakeholders have no benchmarks or criteria to hold the healthcare system accountable or to measure quality of care. We identified several targets that could be incorporated into guidance for improved quality of care, including setting of national stillbirth rate targets.

5. Most high- and upper-middle income countries do not have guidelines around bereavement care, or mechanisms in place for reduction of stigma.

Stigma enables the perpetuation of common myths around stillbirth, such as the belief that stillbirth is inevitable. Putting mechanisms in place to reduce stigma and developing guidelines for appropriate bereavement care should be a priority.

**Supplementary Information:**

The online version contains supplementary material available at 10.1186/s12884-023-05765-5.

## Background

Late gestation stillbirth, defined by the World Health Organization (WHO) as the death of a baby from 28 weeks gestation up to or during birth [1], remains a huge burden worldwide, with an estimated 1.9 million babies stillborn in 2021 [2]. Stillbirth results in profound and long-lasting psychosocial and economic effects for affected families as well as those who provide healthcare for them [3]. Though the burden of this tragedy is largely borne by low-income countries (2019 stillbirth rate [SBR] 22.7/1,000 total births) [4, 5], stillbirth remains a burden in high-income countries (HIC) as well (2019 SBR 3.0/1,000 total births) [5, 6], where at least one-third of stillbirths are potentially preventable [7].

Improvements have been observed in HIC, with a 24.4% decrease in SBR between 2000 and 2019 [5]. Nonetheless, static or even increasing SBRs and a wide variation of rates across HIC show that further improvements in stillbirth prevention are possible. Globally, Japan, the Republic of Korea, Denmark and Finland have maintained the lowest rates for a decade, with SBRs remaining below 2.0 stillbirths per 1,000 livebirths for 2021 [2]. However, the 2021 SBR in HIC ranged from 1.6/1,000 (Japan) to 8.7/1,000 (Trinidad and Tobago), with several HIC reporting higher SBRs than some upper-middle income countries. For instance, the SBR in Bosnia and Herzegovina was reported as 2.7/1,000, while this was 2.8/1,000 in Canada and Germany [2]. There are no known plausible biological reasons for differences in SBRs across HIC, therefore between-country disparities are likely due to other factors, such as national social and political characteristics. Substantial variation in SBRs within as well as between HIC is also present. Just as in low- and middle-income countries, women in HIC experience inequity in stillbirth and other birth outcomes depending upon their socio-economic status, geographic location, and ethnicity, race, or Indigenous status [8–13]. The need to reduce such disparities in SBRs has been recognized as a priority by two Lancet Ending Preventable Stillbirths (EPS) series [14, 15].

A global target to end preventable stillbirths, endorsed by 194 countries at the World Health Assembly in 2014 in resolution WHA67.21 titled the “Every Newborn Action Plan” (ENAP) [16], calls for all countries to reach 12 or fewer stillbirths per 1,000 total births, and to close equity gaps by 2030. The Lancet’s 2016 EPS series included a Call to Action to inspire renewed and focused action for achievement of the global stillbirth target (Table 1) [17]. In response the International Stillbirth Alliance (ISA) Stillbirth Advocacy Working Group (SAWG) developed a scorecard to measure progress against the Call to Action in ENAP target countries in 2018, intended for use by members of civil society including affected parents, researchers and clinicians, to foster transparency, consistency and accountability in stillbirth prevention and care [18]. This EPS Scorecard for Low-Income Countries uses existing indicators and data collection processes by United Nations (UN) agencies and other global organisations where possible, and is updated annually.

**Table 1:** Call to Action to end preventable stillbirths [17]

Mortality targets by 2030 (included in the Every Newborn Action Plan)
1) 12 stillbirths or fewer per 1,000 total births in every country
2) All countries set and meet targets to close equity gaps and use data to track and prevent stillbirths
**Universal health care coverage targets**
3) Family planning: by 2020, 120 million more women and girls with access to contraceptives; by 2030, universal access to sexual and reproductive health-care services and integration of reproductive health into national strategies and programs
4) Antenatal care: by 2030, universal quality of care and comprehensive antenatal care for all women
5) Care during labor and birth: by 2030, effective and respectful intrapartum care to all women in all countries
**Milestones**
6) Every Newborn global and national milestones met by 2020, including the Measurement Improvement Roadmap
7) Respectful care, including bereavement support after a death: by 2020, global consensus on a package of care after a death in pregnancy or childbirth for the affected family, community and caregivers in all settings
8) Reduce stigma: by 2020, all countries to identify mechanisms to reduce stigma associated with stillbirth among all stakeholders, particularly health workers and communities

Subsequently, it was recognized that the Scorecard for Low-Income Countries could be adapted to apply to high- and upper-middle income countries (hereafter “H/UMIC”). In this paper we aim to (1) introduce the Scorecard for EPS in H/UMIC (hereafter “the Scorecard”), (2) report on data from 13 countries for this inaugural (2021) version of the Scorecard, and (3) propose next steps to improve the Scorecard’s utility as a tracking and advocacy tool for ending preventable stillbirths in H/UMIC.

## Methods

### Scorecard adaptation

As described in a companion paper, “Responding to the Lancet’s Call to Action on Ending Preventable Stillbirths: A Global Scorecard” [unpublished results, Leisher SH, et al.], the EPS Scorecard for Low-Income Countries includes a total of 20 indicators to track progress against the eight Call to Action targets. An informal working group was formed in 2019 to adapt the Scorecard for use in H/UMIC, composed of members of the ISA Prevention Working Group and SAWG (see Table 2 for a list of group members).

**Table 2:** Informal working group for adaptation of the Scorecard, and data contributors for inaugural version

Name	Organizational affiliations	Country
**Informal working group members**		
Hannah Blencowe	London School of Hygiene & Tropical Medicine, United Kingdom	United Kingdom
Jillian Cassidy	Asociación Umamanita, Girona, Spain	Spain
Paul Cassidy	Asociación Umamanita, Girona, Spain	Spain
Elizabeth S Draper	MBRRACE-UK, Department of Population Health Sciences, University of Leicester, United Kingdom	United Kingdom
Vicky Flenady	1. NHMRC Centre of Research Excellence in Stillbirth, Mater Research Institute, University of Queensland, Brisbane, Australia 2. International Stillbirth Alliance	Australia
Alexander E P Heazell	Maternal and Fetal Health Research Centre, Division of Developmental Biology and Medicine, School of Medical Sciences, Faculty of Biology, Medicine and Health, University of Manchester, United Kingdom	United Kingdom
Mary Kinney	School of Public Health, University of the Western Cape, Belville, South Africa	South Africa
Susannah Hopkins Leisher	1. NHMRC Centre of Research Excellence in Stillbirth, Mater Research Institute, University of Queensland, Brisbane, Australia 2. International Stillbirth Alliance 3. University of Utah, School of Medicine, United States of America	United States of America
Paula Quigley	International Stillbirth Alliance	Ireland
Claire Storey	International Stillbirth Alliance	United Kingdom
Alfredo Vannacci	PeaRL Perinatal Research Laboratory, CiaoLapo Foundation for Perinatal Health, Department of Neurosciences, Psychology, Drug Research and Child Health, University of Florence, Florence, Italy	Italy
**The EPS in High-Resource Countries Scorecard Collaboration Group (data contributors)**		
Paul Corcoran	National Perinatal Epidemiology Centre, Department of Obstetrics and Gynaecology, University College Cork, Ireland	Ireland
Robin Cronin	1. Department of Obstetrics and Gynecology, University of Auckland, New Zealand 2. Women’s Health, Te Whatu Ora Health New Zealand Counties Manukau, New Zealand	New Zealand
Jan Jaap Erwich	Department of Obstetrics, University Medical Centre Groningen, University of Groningen, Groningen, the Netherlands	The Netherlands
Mika Gissler	1. THL Finnish Institute for Health and Welfare, Department of Knowledge Brokers, Helsinki, Finland 2. University of Turku, Research Centre for Child Psychiatry and Invest Research Flagship, Turku, Finland 3. Karolinska Institutet, Department of Molecular Medicine and Surgery, Stockholm, Sweden 4. Region Stockholm, Academic Primary Health Care Centre, Stockholm, Sweden	Finland
Sanne Gordijn	Department of Obstetrics, University Medical Centre Groningen, University of Groningen, Groningen, the Netherlands	The Netherlands
Guilherme Ramires de Jesús	Department of Obstetrics, Universidade do Estado do Rio de Janeiro	Brazil
Jeannette Klimont	Statistics Austria, Austria	Austria
Aline Lecomte	Department of Precision Health, Luxembourg Institute of Health, Luxembourg	Luxembourg
Marzia Loghi	Directorate for Social Statistics and Welfare, Italian Statistical Institute (ISTAT), Rome, Italy	Italy
Margaret Murphy	School of Nursing and Midwifery, Pregnancy Loss Research Group, University College Cork, Ireland	Ireland
Urelija Rodin	Croatian Institute of Public Health, Head of Department for Research and Monitoring of Maternal and Preschool Healthcare and University of Zagreb, School of Medicine, Andrija Štampar School of Public Health, Zagreb, Croatia	Croatia
Guy Weber	Department for epidemiology and statistics, Directorate of Health, Luxembourg	Luxembourg
Lindsey Wimmer	Star Legacy Foundation	United States of America

The group examined each of the 20 original indicators and proposed one or more new/adapted indicators that might be useful and appropriate in resource-rich settings. For this inaugural edition of the Scorecard, resource-rich settings were considered to include all high- and upper-middle income countries as identified by the World Bank in 2021 [19]. Ultimately, 23 indicators based on 27 data points were selected for inclusion in the Scorecard’s 2021 inaugural edition (see Tables 3 and 4). The 23 indicators were combined into five groups: stillbirth rates (two indicators, using both national and international definitions), related pregnancy outcomes (six indicators, including early neonatal death [ENND], late neonatal death [LNND], preterm birth [PTB] and maternal mortality [MMR]), equity (four indicators) and quality (11 indicators). See Additional file 1 for definitions of each indicator. The Scorecard was pre-tested prior to data collection and analysis, by using country data from Australia and the United Kingdom (UK) to identify gaps in the indicators and to check for user understanding.

**Table 3:** Mapping of data points to indicators

23 Indicators	27 Data points	Indicators Global Scorecard
**Stillbirth rates**		
1.1 SBR 28 weeks or more	1.1 Percent of HIC that have achieved the global stillbirth rate target of 12 or fewer stillbirths (at 28 + weeks) per 1000 total births	1.1 Countries with newborn plan
	1.6 Percent of HIC that have achieved a 28 + week stillbirth rate target of 2 or less stillbirths per 1000 births	1.3 Countries achieved stillbirth rate global target
1.2 SBR national definition	1.2 Percent of HIC with a single national definition of stillbirth	
	1.3 National stillbirth rate	
**Other pregnancy outcomes**		
2.1 ENND rate	1.7 Early neonatal death rate	
2.2 LNND rate	1.8 Late neonatal death rate	
2.3 PTB rate	2.6 Preterm birth rate (total live births at < 37 weeks) per 1000 births	
2.4 MMR	2.9 Maternal mortality rate	4.3 Quality of intrapartum care
2.5 Adolescent pregnancies	2.2 Proportion of pregnancies among adolescent females (USHP2020 FP-8)	2.2 Percentage demand for contraception satisfied
2.6 Planned pregnancies	2.1 Proportion of pregnancies that are planned (USHP2020 FP-1)	2.1 Additional users of modern methods of contraception
**Equity**		
3.1 SBR equity ratio	1.11 Percent of HIC whose stillbirth equity ratio equals 1	1.6 Countries reporting subnational SBRs
3.2 Early and adequate ANC	2.5 Proportion of pregnant women who receive early and adequate prenatal care (USHP2020 MICH10)	3.2 4 + Antenatal care visits
3.3 Early and adequate ANC (among disadvantaged)	2.8 Ratio of pregnant women in disadvantaged to advantaged group with early and adequate prenatal care	3.3 Quality of antenatal care
3.4 Early and adequate ANC equity ratio	2.7 Ratio of pregnant women in lowest 20% wealth bracket to highest 20% wealth bracket with early and adequate prenatal care	
	2.8 Ratio of pregnant women in disadvantaged to advantaged group with early and adequate prenatal care	
**Quality targets**		
4.1 Universal maternity care	2.3 Percent of HIC with universal health care	2.3 Countries with reproductive health plan
4.2 National perinatal audit program	3.1 Percent of HIC with a national perinatal audit program	5.2 Perinatal death review systems
4.3 Adequate perinatal pathologists	3.2 Percent of HIC with a training program for perinatal pathologists	
4.4 Mechanisms for national collection of SB data	3.3 Percent of HIC with a national stillbirth data collection mechanism	
4.5 Government-funded research programs	3.4 Percent of HIC with a national program of research on stillbirth	5.3 Research focusing on stillbirths planned by country
4.6 Classification system	1.12 Percent of HIC using a single classification system at national level to collect data on causes of stillbirths	
4.7 National guidelines bereavement care	3.5 Percent of HIC with national perinatal bereavement care guidelines	5.4 Global consensus on respectful care after stillbirth
4.8 Identified mechanisms for stigma reduction	3.6 Percent of HIC that have identified mechanisms to reduce stigma associated with stillbirth	5.5 National process for stigma reduction
4.9 SBR target	1.4 Percent of HIC that have a public health plan that includes a national stillbirth rate target	1.2 Countries with stillbirth rate target
	1.5 Percent of HIC that have met their own national stillbirth rate target (if any)	
4.10 SBR equity target	1.9 Percent of HIC with a public health plan that includes at least one subnational stillbirth rate equity target	1.4 Countries with subnational newborn plan
	1.10 Percent of HIC that have met their own subnational stillbirth rate equity target(s) (if any)	1.5 Countries with stillbirth rate equity target
4.11 ANC quality target	2.4 Percent of HIC with a quality aim for prenatal/antenatal care	3.1 Global standards for antenatal care

*Abbreviations*: *ANC* Antenatal care, *ENND* Early neonatal death, *HIC* High-income countries, *LNND* Late neonatal death, *MMR* Maternal mortality rate, *PTB* Preterm birth, *SB* Stillbirth, *SBR* Stillbirth rate

**Table 4:** Twenty-three indicators for the Scorecard’s 2021 inaugural edition

Stillbirth rates
1.1 Stillbirth rate using global 28 weeks or more definition
1.2 Stillbirth rate using national definition, if any
**Other pregnancy outcomes**
2.1 Early neonatal death rate
2.2 Late neonatal death rate
2.3 Preterm birth rate
2.4 Maternal mortality ratio
2.5 Rate of adolescent pregnancies
2.6 Rate of planned pregnancies
**Equity**
3.1 Stillbirth Equity Ratio
3.2 Rate of early and adequate antenatal care
3.3 Rate of early and adequate antenatal care among disadvantaged subgroup
3.4 Early and adequate antenatal care equity ratio
**Quality** (presence or absence)
4.1 Universal maternity care
4.2 National perinatal audit program
4.3 Adequate perinatal pathologists
4.4 Mechanisms for national collection of stillbirth data
4.5 Government-funded stillbirth research program
4.6 Classification system for causes of stillbirth
4.7 National guidelines for bereavement care
4.8 Identified mechanisms for stillbirth-related stigma reduction
4.9 Stillbirth rate target
4.10 Stillbirth equity target
4.11 Antenatal care quality target

In this Scorecard we introduce the ‘Stillbirth Equity Ratio’ (SER). The SER is calculated by dividing the SBR of the most disadvantaged group by the SBR of the most advantaged group, where disadvantage and advantage are as defined by each country individually. A SER of 1.0 indicates stillbirth rate equity (identical SBRs in both most and least advantaged groups), while a SER exceeding 1.0 indicates inequity. We additionally included an equity ratio for early and adequate antenatal care (using country definitions of “early and adequate”). Finally, we included 11 quality indicators, such as availability of universal maternity care, national perinatal audit systems and national guidelines for bereavement care, each recorded as being either present or absent (see Table 4 for the complete list).

### Data collection and analysis

For this inaugural version of the Scorecard, contacts in 44 countries (36 high- and eight upper-middle income countries [19]) were invited to contribute national data. This included all members of the Lancet Stillbirths in High-Income Countries Investigator Group (see list of authors from 43 institutions in 14 countries, mainly universities, non-profit organizations and research institutes, available at [20]) as well as ISA working group members. Contacts were selected based on their experience with or connection to stillbirth data and research within their respective countries. Data were therefore not collected directly from governments. Several reminders were sent out, and the Scorecard data collection form was shared within the ISA SAWG membership with the aim to identify additional country contacts. Data collection took place between March 2020 and July 2021. Each contact was asked to supply the following information (see Table 2 for a list of data contributors and Additional file 1 for data collection form):

1. The most recent data for each indicator.
2. The time period for the data provided.
3. Definitions for all terms used.
4. Source(s) for the data, including hyperlinks.
5. Any contextualizing, qualifying or additional information that might be useful for data interpretation, as well as comments on data limitations.

Country data were collated and categorized, summary statistics produced, and similarities and differences between countries described. All data are available in individual raw form, supplied as supplementary information files. Rates of perinatal-related mortality (including stillbirth, ENND and LNND) were compared between countries. There is no globally agreed SBR target for high-resource settings. We therefore considered a SBR of 2.0/1,000 total births, approximately equal to the lowest known national rate in 2021 to be a reasonable benchmark [2], and compared national SBRs to this. To further explore inequity in SBRs, we investigated the correlation between gross domestic product (GDP) per capita and ≥ 28 week gestation SBRs and compared these between countries [21]. We repeated this analysis for the Gini Index (a measure of income inequality) and ≥ 28 week SBRs [22]. Finally, the percentage of all eleven quality targets reported as “present” was calculated for each country, and mapped to allow for between-country comparison.

To demonstrate how the Scorecard could be used to measure progress over time within countries, we also compared data from two time periods for four countries that provided updated data to the group (Australia, New Zealand [NZ], the UK, and Spain). For each indicator we specified whether data from the more recent time period showed improvement or worsening/no progress compared with the earlier time period, or whether a comparison was not possible (due to, for instance, lack of data for the earlier time period).

## Results

Data were received from 13 out of 44 country contacts (30%): Australia, Austria, Brazil, Croatia, Finland, Ireland, Italy, Luxembourg, the Netherlands, NZ, Spain, the UK and the United States of America (USA). Stillbirths in these countries account for 47% and 8% of stillbirths in all high- and upper-middle income countries, respectively [5]. Data for 15 of the 23 indicators (65%) were provided by all 13 country contacts. The lowest responses were for indicators on planned pregnancies, SERs and early and adequate antenatal care. See Additional file 2 for data from each country and Additional file 3 for country-specific definitions for each indicator.

**Key message 1: Wide disparities in stillbirth rates exist between and within high- and upper-middle income countries, indicating that further reduction in stillbirth rates is possible.**

**Fig. 1:**
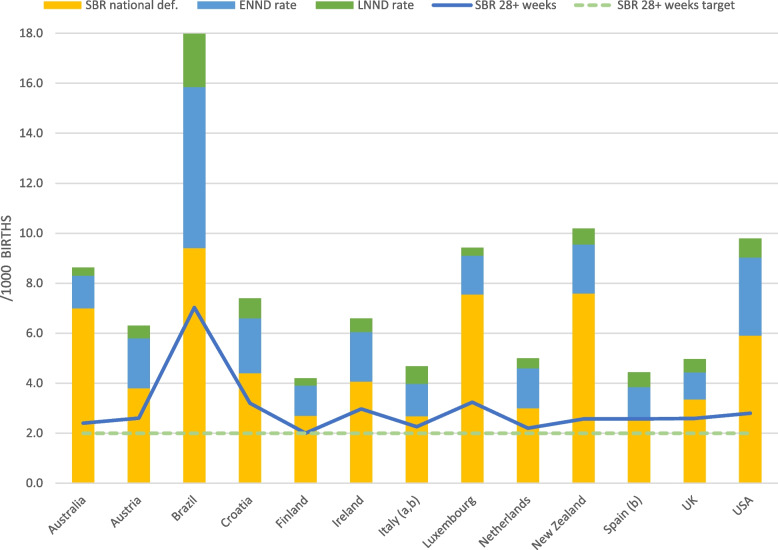
Perinatal-related mortality rates using national definitions, against a 28 + weeks stillbirth rate benchmark. *Notes to figure*: See Additional file 3 for country-specific definitions. ^a ^Stillbirth rates are for 25 + weeks gestation (data missing for 28 + weeks). ^b^ Data from Spain and Italy may be inaccurate due to data quality issues, such as missing data and underreporting. In the case of the Spanish 2019 dataset (National Institute of Statistics, fetal deaths), gestational age data was missing for 12% of cases. Based on a previously conducted analysis of the 2015 dataset, 84% of cases with missing data on gestational age were replaced with a gestational age ≥ 28 weeks. *Abbreviations*: ENND, early neonatal death; LNND, late neonatal death; SBR, stillbirth rate

The SBR at ≥ 28 weeks gestation ranged from 2.0 (Finland) to 7.0 (Brazil) per 1,000 total births, indicating that the global SBR target of 12 or fewer stillbirths per 1,000 total births has been met by all included countries. The SBR according to national definitions ranged more widely, from 2.7 (Finland and Italy) to 9.4 (Brazil) per 1,000 total births, where Italy records stillbirths from 25 + 5 weeks gestation and Brazil and Finland from 22 weeks gestation. There were also wide variation in other perinatal outcome data. While the ENND rate fell between 1.1 (UK) and 2.2 (Croatia) per 1,000 livebirths in 11 countries, there were two outliers: the USA (3.1) and Brazil (6.5). LNND rates were at or under 0.8 per 1,000 livebirths for all H/UMIC except Brazil (2.1) (see Fig. 1). Most countries reported a PTB rate between 5.5% (Finland) and 8.7% (Australia). Brazil and the USA presented as outliers (PTB rates of 11.0% and 10.0%, respectively), which did not correspond to gestational age cut-offs for birth definitions.

**Key message 2: Numerous disparate national stillbirth definitions are used in high- and upper-middle income countries, limiting comparisons necessary to drive change.**

All countries had a national definition for stillbirth that included deaths at earlier gestations, in comparison to the global definition used by WHO and other UN agencies (≥ 28 weeks gestation). Reported definitions included one or more of three key characteristics (see additional file 3):

- Gestational age: 12 countries defined stillbirth as death from 20–26 weeks gestation onwards. Three countries counted from 20 weeks gestation, four countries from 22 weeks gestation and five countries from 24 weeks gestation.
- Birthweight: one country (Austria) defined stillbirth as ≥ 500 g, while all other countries used birthweight as a surrogate when gestational age is missing.
- Inclusion of induced termination of pregnancy (TOP): at least four countries included terminations as part of stillbirth data, five countries excluded TOP (possibly due to legal restrictions for TOP after a certain gestational age) and four countries did not provide this information.

**Key message 3: Data on key risk factors and equity in stillbirth rates are limited, however, underline the need for increased focus on the most affected communities.**

Important risk factors for stillbirth include unplanned and adolescent pregnancies. Only five countries provided data on the proportion of pregnancies that were planned. Rates were comparable across Brazil, NZ, the UK and USA, at 40–55%; however, the proportion of planned pregnancies was significantly higher in the Netherlands, at 80%. Data on adolescent pregnancies were provided by all 13 countries, although there were differences in definitions: most countries included births to women under 20 years old, while some counted livebirths only, and others included total conceptions. Italy and the Netherlands had the lowest rates of adolescent pregnancy (0.8%), whilst the highest rates were reported in NZ and the USA (4.3%) and Brazil (14.5%).

Disadvantaged groups were defined as the Indigenous peoples of Australia, Brazil, NZ and the USA; people of certain ethnicities (Pacific peoples in NZ and the USA, Black ethnicity in the UK); and immigrant groups in Finland and Italy. Data to calculate SERs were provided by seven out of 13 H/UMIC (see Table 5). All had SERs greater than 1.0, indicating inequity in SBRs within each country. Inequity was lowest in NZ with a SER of 1.2 (measuring disadvantage by poverty and ethnicity), followed by Finland (1.3, migrant status), Australia (1.5, poverty) and Brazil (1.5, geographical regions). Three countries had a SER at or over 2.0, with the largest equity gap between Asian/Pacific Islanders and Black/African-Americans in the USA, with a SER of 2.4.

**Table 5:**
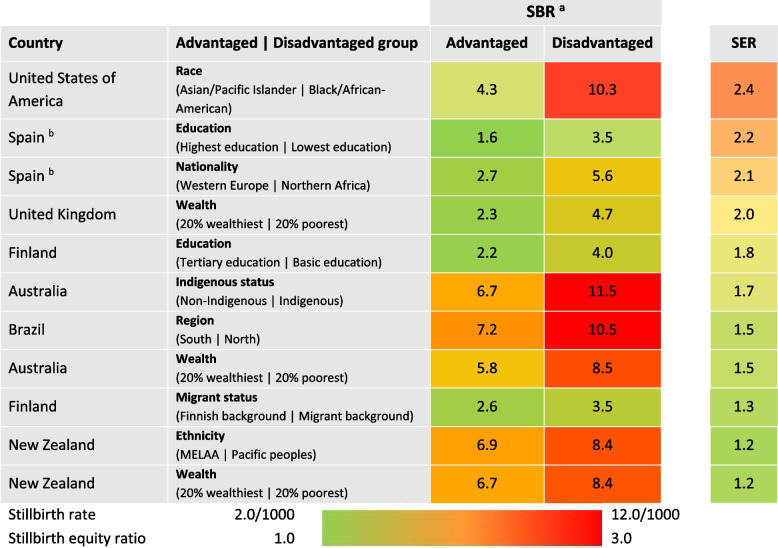
Stillbirth Equity Ratios (SER) for seven countries with available data, ordered by SER

*Abbreviations: MELAA* Middle Eastern, Latin American and African, *SBR* Stillbirth rate, *SER* Stillbirth equity ratio

^a^National stillbirth rates used; see Additional file 3 for definitions

^b^Data from Spain may be inaccurate due to data quality issues, such as missing data and underreporting. In the case of the Spanish 2019 dataset (National Institute of Statistics, fetal deaths), gestational age data was missing for 12% of cases. Based on a previously conducted analysis of the 2015 dataset, 84% of cases with missing data on gestational age were replaced with a gestational age ≥ 28 weeks

**Fig. 2:**
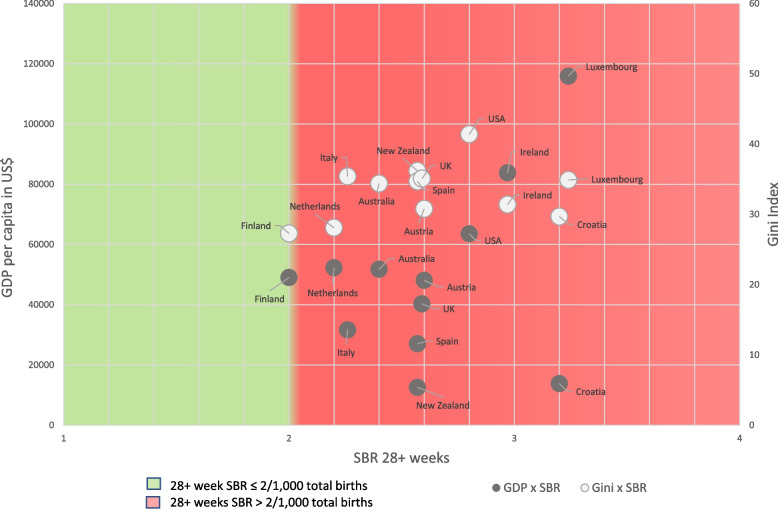
Scatterplot: GDP per capita in $US (2020) and Gini Index by 28 + weeks stillbirth rate. *Notes to figure*: Sources: GDP per capita in US$: World Bank [21]; Gini Index (a measure of income equality): World Bank [22]; except for New Zealand (worldpopulationreview.com) [23]. As an outlier, Brazil was not presented in this figure (SBR 28 + weeks = 7.0; GDP = 6789; Gini = 53.4). ^a^ Italy stillbirth rates are for 25 + weeks gestation (data missing for 28 + week). ^b^ In Luxembourg, where a significant proportion of GDP refers to repatriated profits and thus is not available for national consumption, Gross National Income may be a more meaningful measure than GDP. However, for consistency, GDP was used as the denominator for all countries. *Abbreviations*: GDP, Gross Domestic Product; SBR, Stillbirth Rate

There was no linear correlation between GDP per capita and SBRs, using the ≥ 28 weeks definition for stillbirth (*r* = -0.30, *p* = 0.69, Fig. 2). A positive linear correlation was observed between the Gini Index and SBRs ≥ 28 weeks (*r* = 0.85, *p* < 0.01), however, this did not remain after exclusion of Brazil as an outlier (*r* = 0.34, *p* = 0.40).

The ANC equity ratio was calculated from data supplied by eight country contacts. Italy reported an ANC equity ratio of 1.0, indicating an equal proportion of early and adequate ANC for all women, regardless of disadvantage status (measured as immigrant status). Australia, Finland and the Netherlands performed second best, with an ANC equity ratio slightly greater than 1.0 (1.1), followed by the UK and the USA (1.2), NZ and Brazil (1.5).

**Key message 4: Most high- and upper-middle income countries lack guidelines and targets on key areas critical for stillbirth prevention and care after stillbirth, including national stillbirth rate targets.**

**Fig. 3:**
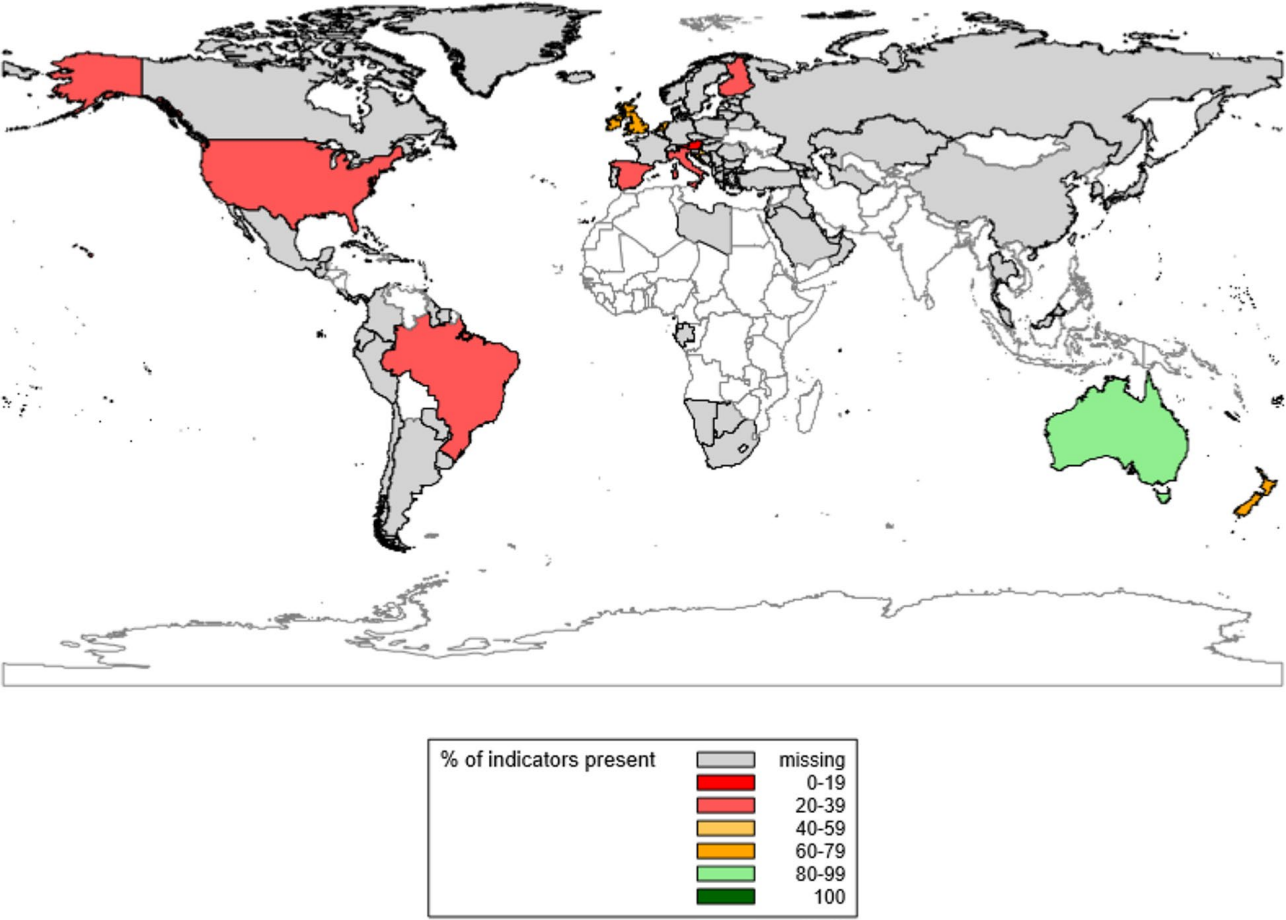
Percentage of the 11 quality targets reported as ‘present’ for 13 countries globally. *Notes to figure*: Quality indicators are 4.1 to 4.11 in Additional file 2. Quality indicators with missing data counted as ‘absent’ in total % calculations. Australia = 91%; Austria = 18%; Brazil = 27%; Croatia = 46%; Finland = 36%; Ireland = 73%; Italy = 36%; Luxembourg = 55%; the Netherlands = 64%; New Zealand = 64%; Spain = 36%; United Kingdom (UK) = 73%; United States of America (USA) = 27%

**Fig. 4:**
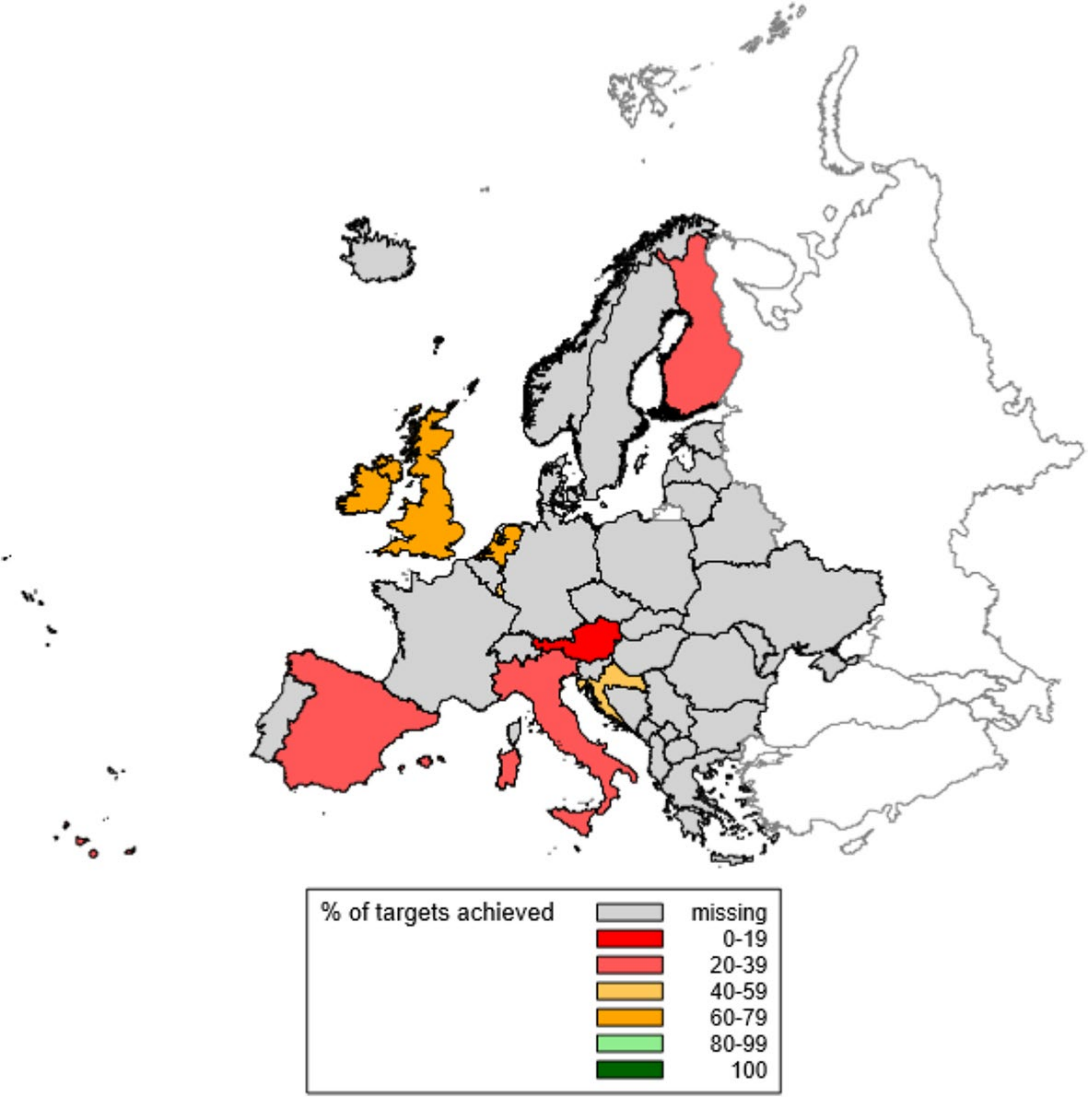
Percentage of the 11 quality targets reported as ‘present’ for eight European countries. *Notes to figure*: Quality indicators are 4.1 to 4.11 in Additional file 2. Quality indicators with missing data counted as ‘absent’ in total % calculations. Austria = 18%; Croatia = 46%; Finland = 36%; Ireland = 73%; Italy = 36%; Luxembourg = 55%; the Netherlands = 64%; Spain = 36%; United Kingdom (UK) = 73%

Australia has implemented a higher percentage of the 11 quality targets than the other reporting countries (91%), followed by Ireland and the UK (73%) (see Figs. 3 and 4). The quality indicators most commonly reported as present were: the availability of universal maternity care (12/13 countries; only missing in the USA); having mechanisms in place for national collection of stillbirth data (12/13 countries); having set an ANC quality target (11/13 countries); and the use of a classification system for causes of perinatal mortality (10/13 countries). Three different perinatal death classification systems were reported as being in use: the WHO International Statistical Classification of Diseases and Related Health Problems 10^th^ Revision (ICD-10) in seven countries, the Perinatal Society of Australia and New Zealand Perinatal Death Classification (PSANZ) in two countries, and Causes of Death and Associated Conditions (CODAC) in one country. In Austria the ICD-10 classification system was reported for neonatal deaths, but not for stillbirths. In the Netherlands no single classification system was identified. However, it was reported that at the start of the national perinatal audit program a combination of systems was used (i.e. Wigglesworth, ReCoDe and Tulip), and death classification was subsequently halted after issues were identified with standardized application.

The quality indicators most commonly reported as absent were: having a SBR equity target (only present in Australia); having guidelines for national bereavement care (3/13 countries, see key message 5); having identified a mechanism for reduction of stigma (3/13 countries, key message 5); and having set a national SBR target (3/13 countries: Australia, UK and the USA). In Australia, the ‘National Stillbirth Action and Implementation Plan’ (NSAIP) aims to reduce the SBR after 28 weeks gestation by 20% [24]. Within the UK, England has set an aim to reduce the SBR by 50% by 2025, compared to a 2010 baseline rate [25]. Finally, in the USA, the national ‘Healthy People’ health plan has set a 2030 SBR target of 5.7/1,000 total births [26]. Of countries with government research programs (4/13), Australia has a national research centre: the Centre of Research Excellence in Stillbirth (Stillbirth CRE), funded by the National Health and Medical Research Council.

**Key message 5: Most high- and upper-middle income countries do not have guidelines around bereavement care, or mechanisms in place for reduction of stigma.**

National guidelines for bereavement care after stillbirth were reported as present in three countries (Australia, Ireland and the UK) [27–29]. Identification of mechanisms for stigma reduction was only reported as present in Australia, Ireland and the Netherlands. In Australia this mechanism includes an accepted recommendation by a Select Committee on Stillbirth Research and Education, for the federal government to “develop and implement a national stillbirth public awareness campaign… which aims to demystify stillbirth, educate parents and the general public about the risks of stillbirth, and encourage public conversations about stillbirth as a public health issue” [30]. In Ireland, the reported mechanisms include a national patient-centered maternity care strategy, a survey of women on their experience of maternity care, targeted research, maternity staff training and non-governmental organizations focused in part on stigma reduction. The Netherlands listed several individual initiatives to increase stillbirth awareness with healthcare workers and the general public, although no national collaboration exists.

### Tracking data over time

Overall, using the Scorecard to track progress over time showed limited improvement in stillbirth prevention and care indicators in the four countries for which these data were available, as well as a gap in data related to equity and quality. For Australia, there was improvement for two indicators, and either status quo or worsening for nine indicators (see Additional file 4). For NZ, there was improvement for five indicators and either status quo or worsening for nine indicators. For the UK, there was improvement for five indicators and either status quo or worsening for five indicators, and for Spain, no indicator showed improvement, while there was status quo or worsening for six indicators. The UK was the only one of these four countries whose SBR improved (decreased) over time, yet in the same time period its SER worsened (increased). We were unable to determine change over time for one indicator in Australia, two indicators in the UK and six indicators in Spain, most of these being equity-related. We could not track progress on any of the 11 quality indicators, as for each country the relevant data were only provided for the second (more recent) year.

## Discussion

We present the inaugural (2021) version of the Scorecard for Ending Preventable Stillbirths in High- and Upper-Middle Income Countries along with data from 13 countries, representing 47% and 8% of all stillbirths among high- and upper-middle income countries respectively [5]. Importantly, the scorecard shows that wide disparities persist between and within countries. This work has highlighted some of the important data challenges that need to be addressed to better understand these disparities, and inform commensurate investments and programmatic action to close these. Differences in definitions of stillbirth and related perinatal outcomes continue to limit comparability between settings, and data on important risk factors are frequently lacking. However, where data are available, context-specific relevant data disaggregation can provide a useful tool for tracking and accountability towards closing equity gaps. The Scorecard also identifies gaps in policies, guidelines and targets on key areas required for effective stillbirth prevention and care, such as a lack of SBR targets and quality-related data for stillbirth prevention and bereavement care found in the majority of countries included. The myth that stillbirths are not preventable [31], is contradicted by the data presented here, including both variability in SBRs across H/UMIC and improvements over time in some H/UMIC, showing that a reduction in SBRs to match that of the best-performing countries globally is not only necessary, but possible. This notion is further supported by a retrospective audit of late gestation perinatal deaths in Australia, which revealed that a large proportion of deaths was associated with suboptimal care [32]. A MBRRACE-UK (Mothers and Babies: Reducing Risk Through Audit and Confidential Enquiries – United Kingdom) perinatal confidential enquiry is currently investigating the quality of care provision in the UK [33].

A core component of the Lancet EPS series Call to Action was for all countries to set and meet targets to close equity gaps in SBRs, and to use data to track and prevent these stillbirths [17]. Six years later, the Scorecard shows that equity gaps for stillbirths in H/UMIC persist. In the Australian setting, socially and economically disadvantaged groups such as Aboriginal and Torres Strait Islander peoples, other ethnic populations and rural and remote groups experience approximately twice the rate of stillbirth compared with the Australian average [13]. In the USA, racial disparities in stillbirth include a two-fold higher SBR among Black ethnicities as compared to White women [34]. In the UK, ethnic inequalities play a key role in stillbirth inequity [35]: the 2020 SBR among Black African babies was 7.8 per 1,000 total births, compared with 3.4 stillbirths per 1,000 total births for babies of White ethnicity [33]. The latest MBRRACE-UK Perinatal Mortality Surveillance Report (2022) highlighted the combined impact of deprivation and ethnicity on SBRs, with rates ranging from 2.8 to 8.1 per 1,000 total births depending on these characteristics [33]. Other HIC such as NZ and Spain experience similar inequalities, unique to their own settings [12, 36]. Australia has currently set a SBR equity target in the NSAIP, aiming for SBRs among women who live in rural and remote or socially disadvantaged areas, or are younger than 20 years, that are equal to those in the general population [24].

Of the 13 countries whose data is presented in the Scorecard, Australia is the first to have a government-led call for a reduction in stillbirth disparities between population groups. As with stillbirths in the population at large, stillbirths among disadvantaged groups are often preventable, but further action is needed to remove equity gaps [37]. Several successful interventions are known. The implementation of a culturally safe, evidence-based model of care for Aboriginal and Torres Strait Islander pregnant women in Australia (Birthing on Country service) resulted in significant improvements in antenatal care attendance and preterm birth rates [38], which are both important risk factors for stillbirth. The MAMAACT intervention in Denmark [39], and the MAMTA Child and Maternal Health Program for Black and Minority Ethnic Women in Coventry, UK [40], are two other examples of educational programs designed to improve maternal health and perinatal outcomes among ethnic populations which have also had success. More emphasis on public awareness campaigns for stigma reduction and education with a focus on disadvantaged populations may be helpful, including evaluation of such programs.

Another well-known issue highlighted by the Scorecard is the lack of comparability of data, due to differences in definitions for stillbirth and related perinatal outcomes between HIC, as well as the lack of a single classification system for cause of death and contributing factors [41, 42]. This reduces our ability to understand where progress is being made and to identify roadblocks. For instance, a slowing rate of SBR reduction in some countries [7], or an actual increase in SBRs at earlier gestations in others [20, 43], may be driven in part by the inclusion of late pregnancy terminations in stillbirth data [44]. Varying definitions for stillbirth may be responsible for at least some of the variation in SBRs between HIC, although a study by Zeitlin et al. (2019) on SBRs in 31 European countries using 2015 Euro-Peristat data found that variation could not be explained by differences in reporting practices alone, as 28-week stillbirth rates varied from < 2.3/1,000 total births (Cyprus, Iceland, Denmark, Finland and the Netherlands) to > 3.5/1,000 total births (Slovakia, Romania, Hungary and Bulgaria) [45]. The common use of a 28-week gestational age cut-off for SBRs, while addressing data comparability issues, underestimates the real burden in most HIC where a significant proportion of stillbirths (35% to 50%, depending on definitions) occur between 20 and 27 completed weeks gestation [20]. Noncomparability of data on causes and conditions associated with stillbirth could be resolved by the introduction and uptake of an international classification system. The ISA Prevention Working Group, in partnership with the Stillbirth CRE, is developing a standardized, high-quality classification system for conditions associated with stillbirth and neonatal death for use in data-rich settings [46], in alignment with recommendations from the WHO guidelines for perinatal mortality, that would meet this need [47].

Data for the 23 indicators in this inaugural version of the Scorecard were collected between 2011 and 2020, suggesting that what matters most for stillbirth prevention and care—not only rates, but also factors such as the numbers of adolescent pregnancies and perinatal pathologists—is not tracked consistently. Stillbirth prevention is included in ENAP and the UN Global Strategy for Women’s, Children’s and Adolescents’ Health 2016–30, but was excluded from the Sustainable Development Goals. Global monitoring of SBR trends remains limited and challenged by data quality and other roadblocks [5]. We should therefore continue to advocate for the inclusion of stillbirths in routine perinatal data collection to highlight the global burden [5]. Failing to collect and report data on stillbirths and their risk factors will have a significantly greater impact on population groups whose stillbirth burden is already disproportionately greater. The unforeseen global outbreak of Covid-19 has had a significant impact on stillbirth risk [48, 49], further emphasizing the importance of having appropriate stillbirth reporting strategies and systems in place [50].

Interventions and investigations into stillbirth risk factors and causes are making important strides in reducing national SBRs. Bundles of care for stillbirth prevention implemented in Australia [51, 52], the UK [53], and Scotland [54], have the potential to reduce SBRs and should be adapted and expanded globally. High-quality perinatal mortality audits are essential for continued learning on causes of stillbirth and the identification of risk factors [20]. However, previous research including for the Lancet EPS series has highlighted that very few H/UMIC have a national perinatal audit system, aligned with our finding that audit systems were lacking in about half of the 13 included countries [20, 55]. Australia’s Improving Perinatal Mortality Review and Outcomes Via Education (IMPROVE) educational program [56], is one promising approach to address this challenge. IMPROVE aims to support clinicians in best practice care for women and families after perinatal death, including investigation and audit; the program has been well received in Australia and is available elsewhere throughout ISA [56].

### Strengths and limitations

Over the past decade a few studies have compared national SBRs [5, 20, 57], and there are several comparison tools for SBRs and other related indicators for stillbirth prevention, such as the data visualization tools available on the Healthy Newborn Network website [58]. However, this inaugural (2021) version of the H/UMIC Scorecard is the first tool created to measure progress on stillbirth prevention and bereavement care in H/UMIC against the Lancet’s 2016 EPS Call to Action. The Scorecard provides H/UMIC civil society with a tool to foster transparency, consistency and accountability for stillbirth prevention and care at national, subnational and global levels, as well as helping to systematically assess progress and roadblocks over time (both between and within countries) and to promote collaboration in addressing stillbirth. There was also a relatively high coverage for high-income countries stillbirths (47%).

The Scorecard has some limitations. First, despite several attempts to reach potential country contacts, we only succeeded in engaging a limited number for this study. Only 30% of the country contacts we reached out to provided data, and these represent just 10% of all 135 H/UMIC that could potentially use this Scorecard [19]. Thus, the results presented in this paper do not reflect the stillbirth situation in all H/UMIC. One of the major difficulties was finding appropriate stillbirth contacts, which is related to the limited awareness of the stillbirth burden in these countries. Relatedly, it is possible that countries for which individuals responded and provided data for this inaugural version of the Scorecard have greater stillbirth awareness or potentially better stillbirth outcomes compared with other H/UMIC. The average SBR of the 13 included countries in this study was 4.0/1000 total births according to the latest United Nations Inter-agency Group for Child Mortality Estimation report [59]. In contrast, the SBR in the 31 non-responsive countries was on average 5.4/1000 total births. A way to increase the number of H/UMIC tracked by this Scorecard would be to identify point persons or point agencies responsible for stillbirth and related perinatal outcomes at country level, as in done NZ where the Perinatal and Maternal Mortality Review Committee is responsible for collection and reporting of stillbirth data [12].

Second, data were not collected from governments directly, which could limit H/UMIC government acceptance of conclusions drawn from the Scorecard. Potential bias may have also been introduced by our country contacts, due to the subjective nature of some of the indicators in this scorecard. However, pulling data from multiple sources also allowed us to address the fact that some indicator data, such as stillbirth equity data and rates of planned pregnancy, are not routinely tracked in national reporting systems. The fact that SBRs presented in the Scorecard are consistent with 2019 and 2021 data published by the United Nations Inter-agency Group for Child Mortality Estimation additionally increases confidence that the data reported by country contacts are accurate [2, 59].

Third, data quality for some indicators was low. For example, stillbirths in Spain were likely under-reported by as much as 5–10% for stillbirths ≥ 28 weeks gestation and 50% for stillbirths < 28 weeks gestation [60, 61]. Hence, the data presented in this Scorecard represents a minimum SBR, as the SBR in Spain is likely much higher than reported here. Also, the fact that some of the data were up to a decade old, despite our request for the ‘most recent available data’, suggests limitations of data collection or availability that may also affect quality.

### Next steps

First, we propose to further improve the quality of this Scorecard by carrying out a Delphi survey among key stakeholders (including parents), to check our 23 selected indicators, further define them, and identify any additional gaps in relevant data that should be included, as well as adjusting how indicators are reported, tracked and compared over time. Delphi surveys have been a successful tool for stillbirth prevention, such as for the development of a global classification system for causes of perinatal deaths [46]. In the Scorecard, quality indicators are currently reported as ‘present or ‘absent’, and thus do not reflect underlying quality (e.g. of the national stillbirth research program or perinatal pathology cadre). The dichotomous nature of these indicators hence does not allow for nuanced assessment. For example, although the Netherlands currently does not have a separate government-funded stillbirth research program, there are individual funding opportunities for research into adverse obstetric outcomes including stillbirth. To better quantify progress in and between H/UMIC, the current indicators need to be adjusted to increase their utility as measures of quality of stillbirth prevention and care after stillbirth. Similarly, H/UMIC targets for stillbirth and related perinatal outcomes could be set based on feedback from the Delphi survey, to enable benchmarking of country performance. We also aim to develop an indicator for data quality in future versions of the Scorecard. This could be based on a set of standards that assess key factors such as underreporting, data completeness, efficacy of the reporting system and correct differentiation of types of perinatal death. With similar antecedent risk factors and causal pathways leading to ENND, PTB, admission to neonatal care units and other adverse events, future versions of the Scorecard should also include indicators to help assess whether preventing stillbirth increases the incidence of these other outcomes. For instance, measures to reduce stillbirth such as iatrogenic delivery, may result in a larger proportion of early term births (< 39 weeks gestation), which has been associated with several short and long term health consequences in the newborn like respiratory distress, hypoglycemia, jaundice, neurodevelopmental disorders or even neonatal death [62].

Second, high- and upper-middle income countries were selected for this Scorecard using World Bank definitions. However, although neither GDP per capita nor the Gini Index were correlated with ≥ 28 week SBRs (after removing Brazil as an outlier)—which is consistent with previous findings [63]—the Gini Index did seem to be a more sensitive measure of stillbirth risk [64]. Hence, consideration should be given to selection of countries for the Scorecard based on the Gini Index.

Third, the Scorecard was designed as a reporting tool to track progress both between and within H/UMIC, to help identify areas for improvement. The Scorecard indicators are being used in the Australian NSAIP, which has an underlying focus on reducing stillbirth inequity [24]. Measuring progress will also be useful in assessing whether the indicators in the Scorecard are the right ones—whether they make a difference for the stillbirth burden. We aim to present an updated report biannually, and to motivate an increasing number of high- and upper-middle income countries to participate in the Scorecard. Finally, we propose to use the Scorecard to advocate for key changes globally, such as the development of a common definition for stillbirth specific to H/UMIC, to help track progress and increase comparability.

## Conclusions

This inaugural version of the EPS Scorecard for High- and Upper-Middle Income Countries highlights important gaps in data and performance for stillbirth prevention and care after stillbirth. There is wide variation in stillbirth rates and related perinatal outcomes, as well as variation in definitions used. Hence, further improvement in stillbirth prevention is possible, and universal definitions for stillbirth and related perinatal outcomes that are specific to high- and upper-middle income countries should be developed to support this ongoing aim. Data on key risk factors for stillbirth are often missing and equity is not consistently tracked, hampering stillbirth prevention strategies. Finally, most countries lack guidelines and targets in critical areas for stillbirth prevention and care, such as national stillbirth rate targets, mechanisms for reduction of stigma and guidelines for bereavement care. This paper can be considered a renewed Call to Action to end preventable stillbirths in high-resource settings, with an emphasis on reducing inequities for disadvantaged groups. We hope civil society will use the Scorecard to hold countries accountable, enable ongoing assessment of progress, share experiences or effective interventions, and promote collaboration in addressing stillbirth.

## Supplementary Information

**Additional file 1.** The Scorecard’s 2021 inaugural edition used for data collection.

**Additional file 2.** Scorecard for Ending Preventable Stillbirths in High- and Upper-Middle Income Countries: Inaugural version (2021).

**Additional file 3.** Definitions used by the 13 countries whose data are included in the inaugural version (2021).

**Additional file 4.** Using the Scorecard to track progress over time: An example with data from four countries.

## Data Availability

All data generated or analysed during this study are included in this published article and its supplementary information files.

## Abbreviations

ANC: Antenatal care
CODAC: Causes of Death and Associated Conditions
ENAP: Every Newborn Action Plan
ENND: Early neonatal death
EPS: Ending Preventable Stillbirths
GDP: Gross domestic product
HIC: High-income countries
H/UMIC: High- and upper-middle income countries
ICD: International Classification of Diseases
IMPROVE: Improving Perinatal Mortality Review and Outcomes Via Education
ISA: International Stillbirth Alliance
LNND: Late neonatal death
MBRRACE-UK: Mothers and Babies: Reducing Risk Through Audit and Confidential Enquiries – United Kingdom
MELAA: Middle Eastern, Latin American and African
MMR: Maternal mortality ratio
NSAIP: National Stillbirth Action and Implementation Plan
NZ: New Zealand
PSANZ: Perinatal Society of Australia and New Zealand
PTB: Preterm birth
SAWG: Stillbirth Advocacy Working Group
SBR: Stillbirth rate
SER: Stillbirth equity ratio
Stillbirth CRE: Centre of Research Excellence in Stillbirth
TOP: Termination of pregnancy
UK: United Kingdom
UN: United Nations
USA: United States of America
WHO: World Health Organization
